# Exploring Maternal Self-Efficacy of First-Time Mothers among Rural-to-Urban Floating Women: A Quantitative Longitudinal Study in China

**DOI:** 10.3390/ijerph18062793

**Published:** 2021-03-10

**Authors:** Qun Wang, Yao Zhang, Xilin Li, Ziwen Ye, Lingling Huang, Yan Zhang, Xujuan Zheng

**Affiliations:** Health Science Centre, Shenzhen University, Shenzhen 518060, China; qunwang@szu.edu.cn (Q.W.); zhangyao@szu.edu.cn (Y.Z.); xy_l1991@163.com (X.L.); y673817550@126.com (Z.Y.); huanglingling@szu.edu.cn (L.H.); xiahzhang@szu.edu.cn (Y.Z.)

**Keywords:** postpartum women, self-efficacy, floating women, postnatal depression, Mainland China

## Abstract

(1) Background: China has the highest number of rural-to-urban floating women in the world, and the majority of them are of childbearing age. However, few studies have focused on maternal self-efficacy (MSE) for these women. This research aims to explore MSE and its influencing factors for primiparous women among the rural-to-urban floating population in China. (2) Methods: A quantitative longitudinal study was conducted, and primiparous women from the floating population were recruited in China. Face-to-face demographic questionnaires were collected from obstetric wards by the researchers, three days postpartum. The 6-week and 12-week questionnaires, including the Self-efficacy in Infant Care Scale (SICS), the Edinburgh Postnatal Depression Scale (EPDS), and the Postpartum Social Support Scale (PSSS), were sent to participants by wechat or email to measure MSE, postnatal depression, and social support, respectively. The completed 6-week and completed 12-week questionnaires were returned to the researchers. (3) Results: The mean MSE scores at 6 and 12 weeks postpartum were 67.16 (SD = 14.35) and 68.71 (SD = 15.00). The variables of social support, postnatal depression, whether women attended parenting training, baby fussiness, baby health, maternal educational level, occupation, and family income affected MSE at the two time points. (4) Conclusions: Primiparous women among the rural-to-urban floating population had a low MSE level. As a vulnerable and special group, more attention should be paid to the negative parenting status of floating women by health workers and family members. Effective measures must be taken to improve the floating women’s accessibility to parenting training from health services to strengthen their social support and alleviate postpartum depression. Health professionals should be more concerned with floating women with relatively low MSE levels, such as new mothers with lower education, poor working and living conditions, unhealthy babies, and babies with fussy temperaments.

## 1. Introduction

With the rapid development of the economy and urbanization in China, a large portion of the population has moved from the countryside to cities in the last three decades; these people are called the “floating population” or “internal migrants” [1,2]. According to a report on the development of the Chinese floating population, there were approximately 247 million people in the floating population in 2020, more than one-sixth of the total population of China [3], and Shenzhen city had eight million floating people, accounting for more than 60% of the inhabitants. A national survey on the floating population by the Health Commission of China in 2018 said that nearly half of the floating population were female, and the majority of them were of childbearing age [3,4,5].

These rural-to-urban floating women play an important role in economic development by meeting the social needs of the industrialized urban areas of China [4,5,6]. However, there are many healthcare benefits and social welfare only available to local city residents [1]. This denial restrains the access of floating women to the full benefits available to city residents and creates social inequality [7]. Studies have shown that health disparities exist between the female floating population and city residents, owing to the lack of social support and unbalanced allocation of health resources [8,9]. Floating women of childbearing age were reported to have poor utilization of reproductive health services and to use fewer perinatal outpatient health services than resident women in terms of prenatal care and postpartum visits [5,6]. For instance, the utilization of reproductive health (RH) services have been found to be poor in floating women; only a minority of migrant women could access RH policies (39.3%), RH education (36.4%), RH counseling (27.4%), gratis contraceptives (36.0%), and free RH examinations (38.9%) [6]. For example, 5372 married rural-to-urban migrant women, aged 20–34, who delivered a baby, were recruited in a large national survey; it was found that many young rural-to-urban migrant women reported no prenatal care in the first trimester of pregnancy and an inadequate number of prenatal visits during their latest pregnancy [5]. Thus, the well-being of women in the reproductive period has drawn significant attention from researchers [1,6].

Transition to motherhood brings great challenges to women, who need to acquire parenting knowledge and skills, adjust to the new household relationship, and accept the maternal role [10]. Many women find it difficult to manage these physical, social, and psychological challenges in early motherhood [11]. Owing to the lack of previous parenting experience, these problems are particularly prominent for first-time mothers [12]. Research findings [13,14] indicate that primiparous women, confronted with many parenting troubles during infancy, such as negative mother–infant interactions and unsuccessful parenting tasks, are negatively impacted in terms of the well-being of infants and mothers.

As an important indicator of parenting outcomes, maternal self-efficacy (MSE) is the perceived ability of a mother to hold up her organization and performance of various parenting tasks [15]. Women with a high level of MSE are identified to conduct positive parenting [16,17]. Considering MSE’s important impacts, an increasing number of researchers have focused on this domain, and related studies of MSE have been undertaken in various countries of the world [18,19,20,21,22]. However, the critical limitations of previous research on MSE were that most studies were limited to a homogeneous sample of well-educated and employed women, and conflicting evidence of what factors affect MSE has been presented in the literature [13,23,24].

In mainland China, the studies relating to MSE have been limited to only first-time mothers among local city residents [14,24]; few studies have focused on MSE for Chinese floating women. Therefore, this research was conducted to firstly explore MSE and its influencing factors for Chinese primiparous women among the rural-to-urban floating population during the initial postnatal period to fill the research gap.

## 2. Materials and Methods

### 2.1. Study Design

Research of quantitative longitudinal design was conducted to explore MSE and its influencing factors on first-time mothers among the Chinese rural-to-urban floating population in the initial postpartum period.

### 2.2. Setting and Sample

The research was conducted in the obstetric wards of two hospitals in Shenzhen City, Guangdong Province. There are over 4000 annual live births in each study hospital. Recruitment took place from March to December of 2019 via recruiting posters and leaflets. The inclusion criteria were as follows: being a first-time mother with a healthy baby, over 18 years of age; rural-to-urban floating women; being able to respond to the questionnaires in Chinese. Exclusion criteria were women or their children being seriously ill or having died.

A suitable sample size in multivariate analysis would be tenfold of the number of independent variables [25]. In the research, the maximum number of independent variables that could possibly impact MSE would be 20; hence, the appropriate sample size would be 200. According to previous research, an average attrition rate for postnatal women was about 30–45% at one time point by email [14,24,26]. In this study, a 45% loss at follow-up was conservatively estimated at every time point; it means that a 55% response rate at the first time point of follow-up and 30% response rate at the second time point of follow-up, so the predetermined recruited sample size was calculated to be 200/30% = 667.

### 2.3. Measures

A demographic questionnaire was developed to collect participant information of maternal age, marital status, educational level, occupation, family income, mode of birth, whether attended parenting training from health services, baby gender, baby health, and baby fussiness via the women’s self-report. Baby health refers to a baby with a good health status. Baby health scores were reported by their mothers from 0 (very unhealthy) to 100 (very healthy). A higher baby health score indicates a higher level of baby health, rated by the mother. Baby fussiness means a baby with a negative or difficult temperament, which has characteristics such as irritability and low soothability and manageability. Baby fussiness scores were reported by their mothers from 0 (extreme fussy) to 100 (not at all). A higher baby fussiness score indicates a lower level of baby fussiness, rated by the mother.

MSE was measured using the Self-efficacy in Infant Care Scale (SICS) [27], a 46-item scale comprising four dimensions. Each item indicates one parenting task; the higher the score, the higher the self-efficacy. For instance, women were asked to rate their degree of confidence in performing designated tasks/situations; a score ranging from 0 to 100 was assigned, depending on their confidence response from “not confident at all to do it” to “definitely confident I can do it”. The scale is scored by summing the numerical ratings for each task and dividing the result by the number of tasks. The reported internal consistency was 0.96 for the total scale and ranged from 0.86–0.96 for its dimensions [27]. The test–retest reliability coefficient for the total scale was 0.93. The Cronbach’s alpha coefficient of the SICS was 0.94 in the present study sample.

Postnatal depression was measured by the Edinburgh Postnatal Depression Scale (EPDS) [28], which is a 10-item four-point Likert instrument. The sum score of the self-report scale ranges between 0–30, and a higher score indicates a worse health status. The Cronbach’s alpha coefficient of the Chinese version of EPDS was 0.87; the concurrent validity with the Beck Depression Inventory was 0.79 [29]. In this research, the Cronbach’s alpha coefficient of the EPDS was 0.85.

Postnatal social support was assessed using the Postpartum Social Support Scale (PSSS), developed for Chinese women to measure their perceived social support after childbirth [30]. A 20-item 4-point Likert scale was used, and a score ranging from 0 to 3 was assigned, depending on response options of “never”, “rarely”, “sometimes”, and “often”. Its total score is in the range of 0–60, and a higher score means more social support. The Cronbach’s alpha coefficient of the PSSS was 0.89, and the content validity of this tool was 0.90 [30]. The test–retest reliability coefficient for the total scale was 0.92. The Cronbach’s alpha coefficient of the PSSS was 0.90 in the current study.

### 2.4. Data Collection

The demographic questionnaires and participants’ contact details were collected by the researchers face to face in obstetric wards at three days postpartum. The 6-week questionnaires comprised of SICS, EPDS, PSSS, baby health, and baby fussiness, and the 12-week questionnaires, including SICS, EPDS, PSSS, baby health, and baby fussiness, were sent to the participants by wechat or email at 6 and 12 weeks postpartum, respectively. The completed questionnaires were returned to the researchers by wechat or email. In order to improve the response rate, polite wechat reminders were sent to participants one week and one day before and after the 6-week and 12-week postpartum periods, respectively.

### 2.5. Data Analysis

SPSS Statistics 21.0 was used for statistical analysis. Descriptive statistics were conducted to describe the sociodemographic characteristics of floating women by means and standard deviations (SDs), frequencies, and proportions. Multiple linear regression analysis was used to explore the potential factors influencing MSE [25]. In this study, the MSE scores are the dependent variable, and the other variables as independent variables were entered into the multiple linear regression model (a_entry_ = 0.05, a_removal_ = 0.10) to explore the influencing factors of MSE for these primiparous women in the floating population at – and 12 weeks postpartum, respectively. Diagnostic tests for assumptions, including linearity, normality, homoscedasticity, independence, and model specification, were completed, and these assumptions were met for the multiple linear regression models in the analysis [25].

### 2.6. Ethical Considerations

This study was approved by the Research Ethics Committee of Health Science Centre, Shenzhen University. Information sheets were distributed to all eligible women to introduce the purposes and process of this research before recruitment. Informed consent was obtained from every participant before data collection. Participants were informed of their freedom to withdraw at any time and were assured of anonymity through the use of special code numbers to identify them. All collected data were treated anonymously and confidentially.

## 3. Results

### 3.1. Recruitment and Participant Flow

**Figure 1:**
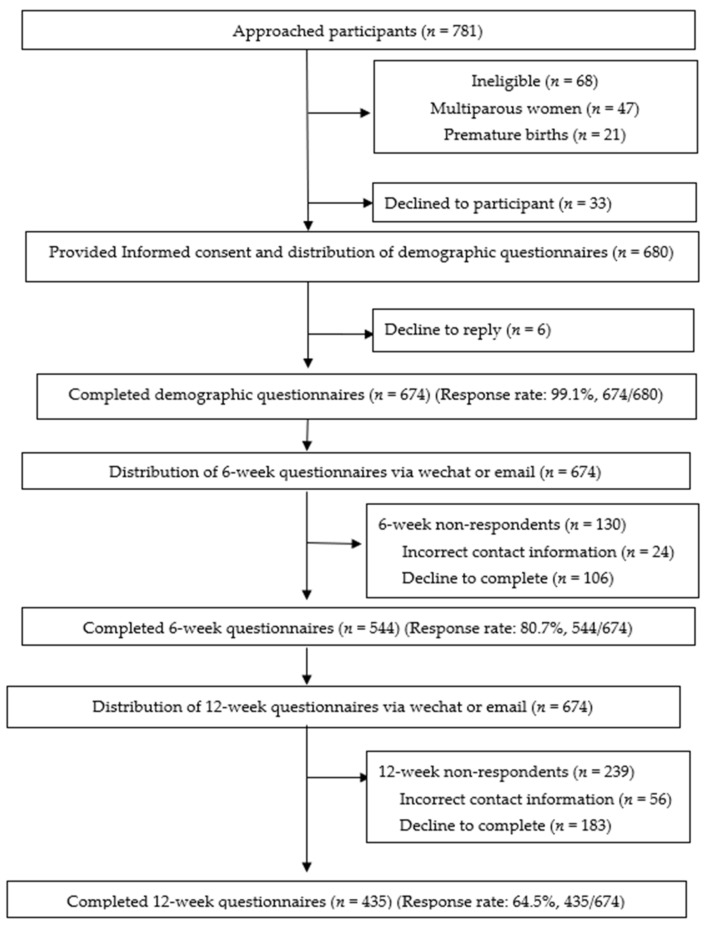
Recruitment and participant flow in this study.

Recruitment and participant flow in the research are shown in Figure 1.

### 3.2. Demographic Characteristics of the Participants

In total, 680 Chinese first-time mothers from the population of floating women were recruited, and 674 women completed the demographic questionnaires. Demographic data revealed the mean age of these floating women was 25.82 (SD = 3.38), and all of them were married. About 1/3 of respondents (27.0%, 182/674) had a university or college degree, and 61.6% (415/674) of them had an unskilled occupation; 42.4% of participants (286/674) had a family income of ¥3000–5000 (USD420–700)/per month per person. These participants had a higher proportion of vaginal delivery (61.4%, 417/674) than cesarean section (20.5%, 138/674), and over half of them (58.2%, 392/674) had a boy. Compared with first-time mothers among city residents (mean age of 27.28, 61.3% with tertiary education, 4.8% having an unskilled job, and 42.1% with a monthly family income more than ¥5000) [14,24], these primiparous women of the floating population had characteristics of younger childbearing age, lower educational levels, and poor living and working environments.

### 3.3. Mean Maternal Self-Efficacy (MSE), Postnatal Depression (PPD), Social Support, Baby Health, and Fussiness Scores at 6 and 12 Weeks Postpartum

The mean MSE scores at 6 and 12 weeks postpartum were 67.16 (SD = 14.35) and 68.71 (SD = 15.00), respectively. The mean social support score increased from 37.04 (SD = 10.15) at 6 weeks postpartum to 38.68 (SD = 10.46) at 12 weeks postpartum, while the mean EPDS score was kept almost stable, from 11.19 (SD = 4.89) at 6 weeks postpartum to 11.18 (SD = 5.34) at 12 weeks postpartum. Mean baby health scores were 83.01 (SD = 14.34) and 83.74 (13.74) at 6 and 12 weeks postpartum, respectively, and the mean baby fussiness scores were 72.83 (SD = 19.06) and 72.66 (SD = 16.72) at the two time points.

### 3.4. Coding Independent Variables in the Multiple Linear Regression Model

According to the requirements of coding, the independent variables in multiple regression models and the continuous independent variables retain the original numerical value as an assigned coding in the model. In relating to dichotomous variables, one variable is coded as 0, and the other is coded as 1. In terms of polytomous independent variables (more than two categories), they were established as dummy variables to represent different comparison groups [25]. To create the set of dummy variables, a reference group or category was firstly decided. Then, dummy variables were created for the remaining groups (excluding the reference group) and coded 1 for participants who were in that group; all others were coded 0. Therefore, there are (k − 1) dummy variables needed for k categories [25]. The coding of independent variables in the regression model is shown in Table 1.

### 3.5. The Regression Results of MSE Scores at Six Weeks Postpartum

The regression results (Table 2) show that seven variables affected MSE (*t*-test, *p* < 0.05); and these variables explained 63.1% of the variance in MSE at six weeks postpartum (adjusted R2 = 0.631, F = 91.225, *p* < 0.001).

### 3.6. The Regression Results of MSE Scores at 12 Weeks Postpartum

At 12 weeks postpartum, 8 variables were statistically significant factors influencing MSE (*t*-test, *p* < 0.05); these variables explained 73.5% of variance in MSE (adjusted R2 = 0.735, F = 134.410, *p* < 0.001; Table 3).

## 4. Discussion

### 4.1. The MSE of First-Time Mothers among Rural-to-Urban Floating Women

In this study, the mean MSE scores of primiparous women in the floating population were 67.16 (SD = 14.35) at six weeks postpartum and 68.71 (SD = 15.00) at 12 weeks postpartum, which are significantly lower than previous findings from first-time mothers of city residents in China, whose MSE scores were 74.92 (SD = 11.05) and 77.78 (SD = 11.13) at the same time points [14,24]. These scores are also obviously lower than the MSE score of 80.02 (SD = 11.70) in a sample of Thai women using the same measurement of SICS [31]. The results indicated that these first-time mothers of floating women had a low MSE level.

There are some reasons that could explain this phenomenon. Firstly, parenting a baby is not only a significant matter for a Chinese mother but also an important issue for the whole family; retired grandparents have more time and will volunteer to help new mothers look after their babies [32]. One survey result of 20,083 seniors showed that 80% of urban elderly females participate in raising grandchildren [33]. These urban grandmothers have rich experiences in child-rearing and easily translate past experiences into current parenting practices for new urban mothers, which could definitely improve these women’s MSE levels [34]. However, many floating women lacked the babies’ grandparents’ involvement in child-rearing because most of their parents live in relatively poor and underdeveloped rural areas and rarely move to urban areas to live together in consideration of the economic burden. Secondly, under China’s longstanding household registration system, these floating women have an inevitably institutional barrier in accessing social welfare and healthcare benefits due to the absence of official local residence and medical insurance [1]. It means that compared with city residents, these rural-to-urban migrants acquire less health care and social support [8,9], which negatively affect their parenting confidence. Thirdly, as a transmigration group between urban and rural areas, floating women have relatively low educational levels and poor living and working environments [35]. Researchers [24,36] found that mothers with lower educational levels, worse occupations, and lower family incomes were prone to having a lower mean MSE score, which is likewise supported by this research finding.

As a vulnerable and special group, the negative parenting status of primiparous women in the floating population should be paid more attention to by the Chinese government, researchers, and health professionals. It is strongly recommended that future effective intervention should be conducted to improve these first-time mothers’ MSE levels, which significantly impacted maternal well-being and child development [16,17].

### 4.2. Factors Influencing MSE for Chinese Floating Women

#### 4.2.1. Social Support

According to the results of this study, social support was the main influencing factor of MSE at the two time points, which is consistent with most prior studies in different countries [21,24,26,36,37]. The result highlighted that women receiving more social support, in their perception, were more likely to have a higher level of MSE.

Some factors probably accounted for this result. Bandura [34] identified four major elements that affected self-efficacy: previous experience, vicarious experience, verbal persuasion, and physiological and emotional states. Social support could influence MSE level through the last three elements. First of all, support providers would give new mothers more vicarious parenting experience through observational learning from other women [16]. For instance, new mothers may observe successful parenting performance from obstetric nurses, midwives, and family members to learn or model their effective parenting behaviors. Secondly, supporters may give first-time mothers wonderful advice, information, and evaluation of child-rearing that positively impact their MSE levels through verbal persuasion [16]. Thirdly, social supporters may supply women with emotional and material support, such as encouragement, concern, financial assistance, and time to help them have a good physiological and emotional recuperation from childbirth, which increased new mothers’ parenting competence.

#### 4.2.2. Postnatal Depression

The study findings identified that postnatal depression was an important factor affecting MSE at 6 and 12 weeks postpartum, which is consistent with previous research [20,24,32,36]. The following evidence may explain how postnatal depression of women negatively influences their MSE levels. One reason is that depressed mothers often suffer from lack of energy, feeling exhausted and sleepy, and the worse physical status makes it difficult for them to bond with and take care of their babies. Furthermore, women with postnatal depression usually experience a persistent feeling of sadness, irritability, guilty conscience, and hopelessness, and these negative mental conditions seriously suppress new mothers’ parenting confidence and capability [38].

#### 4.2.3. Whether Attended Parenting Training from Health Services

In this study, whether floating women attended parenting training impacted their MSE. It highlights that attending parenting training from health services plays a significant role in the improvement of MSE levels for the sample of Chinese floating women by teaching them professional and scientific parenting skills and information.

By comparison, in the research of Zheng et al. [24], this variable did not enter the multiple linear regression model of MSE at the two time points. The reason for the inconsistent results was probably that the sample of Zheng et al. [24] focused on female city residents, and most (63%) of them attended parenting training from hospitals, while only 40% of floating women attended the related training in this research. It likewise confirmed that the floating women have less access to healthcare sources compared with their counterparts in city residents [8,9].

#### 4.2.4. Baby Fussiness and Baby Health

In the present study, baby fussiness affected MSE levels at the two time points. Past research conducted in Western countries [18,39] revealed consistent evidence that new mothers with less fussy and irritable infants were reported to have a higher MSE level. Babies with fussy temperament are characterized by slow adaptability, negative mood, and intense reaction. Thus, taking care of these infants increases the difficulty of parenting tasks and leads to more challenges in the achievement of maternal role expectancy and more unsuccessful parenting experiences [40].

The interesting finding in this study was that baby health only remained in the multiple linear regression model at 12 weeks postpartum but not in the regression model at 6 weeks postpartum. This result implies that compared with other major influencing factors such as postnatal depression and social support, baby health does not have an important effect on MSE in the shorter term. However, with the passage of time, baby health conditions significantly influence maternal physical and emotional status, which impact MSE, according to the theory of Bandura [34]. The result was in accord with other research findings, with a longer follow-up period [24,41,42], that noted that women with a self-reported healthier baby tended to score higher on MSE at three and six months postpartum. For example, some Hong Kong mothers expressed that witnessing their infants’ well-being increased their maternal role competence at six months postpartum [42]. One mother said, “I was quite competent in mothering because my child was healthy and did not have any problem” [42]. Thus, a conclusion can be drawn that baby health affects MSE for rural-to-urban floating women in the longer term, but not in the shorter term.

#### 4.2.5. Maternal Educational Level, Occupation, and Family Income

The other three influencing factors of MSE among floating women in this study were maternal educational level, occupation, and family income at 6 and 12 weeks postpartum. Indeed, these three variables are interrelated and interact with each other [26].

The findings indicate that mothers with a higher educational level have a higher MSE score. Past research [24,43] agrees that women with at least a tertiary education will score higher on MSE than women with a high or middle school degree. The explanation may be that better-educated mothers have a stronger independent learning capability to acquire more parenting information and knowledge than lower-educated mothers [24]. In contrast, the other research has shown that maternal education level was not related to MSE in a sample of Singaporean women [37] and Chinese urban women in Guangdong City [44]. The main reason causing the inconsistent findings that the majority (80%) sample of the two studies had a university degree or higher, and the skewed education level groups may have led to selection bias.

In this study, mothers with professional occupations had the highest MSE scores in comparison with mothers with other work and the unemployed, which is consistent with previous study findings among female city residents in China [24]. Another study undertaken in a Western country [45] had similar results; women with professional jobs had greater MSE than women with casual jobs because women with professional occupations tend to be mothers with better education levels, which positively influences their parenting confidence, as shown by various studies [24,43].

The present study found that higher family income was one predictor of higher MSE scores for these rural-to-urban floating women. The research by Shorey et al. [36], conducted in Singapore, also demonstrated that higher family income was associated with a higher MSE level for women with infants. Mothers with low family income often suffer from financial problems that cause them to be more stressed, depressed, or irritable, which can negatively affect their parenting confidence. However, Zheng et al. [24] found that family income did not correlate with MSE scores of city residents, which is consistent with previous studies undertaken in different countries [26,36,44]. These conflicting results could be attributed to the different family and social backgrounds. For example, 42.4% of participants in this study had a family income of ¥3000–5000 (USD420–700)/per month per person. In contrast, almost 42.5% of the women in the study of Zheng et al. [24] had a family income of more than ¥5000 per month per person, so they probably did not have any severe economic burden that suppresses their maternal confidence.

Compared with city residents, first-time mothers among floating women had the characteristics of lower educational levels and poor living and working environments, which indeed negatively affect their parenting self-efficacy.

In summary, China has the highest number of rural-to-urban floating women in the world, and the majority of them are of childbearing age. However, few studies have focused on the maternal self-efficacy (MSE) of these women. The research aimed to explore MSE and its influencing factors for primiparous women in the rural-to-urban floating population in China. The study findings indicate that first-time mothers in the rural-to-urban floating population have a low MSE level; the variables of social support, postnatal depression, whether women attended parenting training, baby fussiness, baby health, maternal educational level, occupation, and family income were identified to influence MSE in the initial postpartum period. According to the results, the negative parenting status of first-time mothers among rural-to-urban floating women should be the cause of more concern from the Chinese government, researchers, and health professionals. Future tailored intervention is strongly recommended to be designed and conducted to improve these first-time mothers’ MSE levels, which significantly affect the well-being of women and children.

## 5. Conclusions

In this study, we found that primiparous women in the rural-to-urban floating population have a low MSE level in the initial postpartum period. As a vulnerable group, these floating women’s negative parenting status should be paid more attention by researchers and health professionals; this highlights the urgent need for further interventions tailored to the factors affecting MSE. Effective measures must be taken to improve the floating women’s accessibility to parenting training from health services, strengthen their social support, and alleviate postpartum depression. Health professionals and family members should be more concerned with floating women with relatively low MSE levels, such as new mothers with lower education, poor working and living conditions, unhealthy babies, and babies with fussy temperament.

However, several limitations need to be noted. First, the variables in the research were assessed by self-report tools and may have led to a social desirability bias owing to the traditional belief of “domestic shame should not be made public”. Second, this study did not include female city residents as a comparison group for direct comparison. Third, research findings in one city may not generalize to floating women in other regions of China. Fourth, there was no further follow-up after three months postpartum because of time and financial constraints. Therefore, future research could use multiple methods such as interviews and observation to measure variables more accurately. In consideration of regional diversity, this study could be replicated in other cities or regions of China. In order to gain a better understanding of MSE and its influencing factors on floating women, a further longer-term study could be conducted. Furthermore, in the cause of helping rural-to-urban floating women cope better with parenting tasks, future intervention strategies need to be specifically tailored to the identified factors affecting MSE for these women.

## Figures and Tables

**Table 1:** The coding of independent variables in the regression model.

Independent Variables	Methods of Coding
Maternal age	Original numerical value
Education level (* middle school or lower)	
Education level 1	Middle school or lower = 0, High school = 0, University/College = 0, Master’s degree or higher = 1
Education level 2	Middle school or lower = 0, High school = 0, University/College = 1, Master’s degree or higher = 0
Education level 3	Middle school or lower = 0, High school = 1, University/College = 0, Master’s degree or higher = 0
Occupation (* unemployed status)	
Occupation 1	Unemployed status = 0, Professional occupation = 0, Skilled occupation = 0, Unskilled occupation = 1
Occupation 2	Unemployed status = 0, Professional occupation = 0, Skilled occupation = 1, Unskilled occupation = 0
Occupation 3	Unemployed status = 0, Professional occupation = 1, Skilled occupation = 0, Unskilled occupation = 0
Family income (* >5000)	
Family income 1	<3000 = 1, 3001—5000 = 0, >5000 = 0
Family income 2	<3000 = 0, 3001—5000 = 1, >5000 = 0
Mode of birth (* normal vaginal birth)	
Mode of birth 1	Normal vaginal birth = 0, Assisted delivery = 0, Caesarean section = 1
Mode of birth 2	Normal vaginal birth = 0, Assisted delivery = 1, Caesarean section = 0
Whether attended parenting training	No = 0, Yes = 1
Baby gender	Girl = 0, Boy = 1
Baby health scores	Original numerical value
Baby fussiness scores	Original numerical value
Postnatal depression (EPDS scores)	Original numerical value
Social support (PSSS scores)	Original numerical value

* Reference group. EPDS: Edinburgh Postnatal Depression Scale; PSSS: Postpartum Social Support Scale.

**Table 2:** The regression results of maternal self-efficacy (MSE) scores at six weeks postpartum (*n* = 544).

Variables	Unstandardized Coefficients		Standardized Coefficients	t	*p*
	B	Std. Error	Beta (Descending)		
Constant	48.233	2.949		16.36	< 0.001
Social support (PSSS) scores	0.540	0.045	0.382	11.92	< 0.001
EPDS scores	−0.804	0.095	−0.274	−8.47	< 0.001
Family income 2	−6.895	1.099	−0.238	−6.27	<0.001
Baby fussiness scores	0.161	0.022	0.214	7.35	< 0.001
Education 3	4.797	0.940	0.150	5.10	<0.001
Whether attended parenting training	3.700	0.834	0.128	4.39	<0.001
Family income 1	−3.593	1.199	−0.119	−3.00	0.003
Occupation 3	7.787	1.969	0.107	3.95	< 0.001
Education 1	7.335	2.165	0.094	3.39	0.001

F = 91.23, *p* < 0.001, adjusted R2 = 0.631. MSE: maternal self-efficacy; EPDS: Edinburgh Postnatal Depression Scale; PSSS: Postpartum Social Support Scale.

**Table 3:** The regression results of MSE scores at 12 weeks postpartum (*n* = 435).

Variables	Unstandardized Coefficients		Standardized Coefficients	t	*p*
	B	Std. Error	Beta (Descending)		
Constant	34.195	3.413		10.02	<0.001
EPDS scores	−0.997	0.090	−0.355	−11.12	<0.001
PSSS scores	0.444	0.048	0.309	9.20	<0.001
Baby fussiness scores	0.176	0.026	0.196	6.86	<0.001
Baby health scores	0.196	0.032	0.179	6.17	<0.001
Family income 2	−4.897	1.037	−0.163	−4.72	<0.001
Family Income 1	−4.223	1.207	−0.132	−3.50	0.001
Education 2	3.184	0.873	0.105	3.65	<0.001
Whether attended parenting training	2.548	0.814	0.085	3.13	0.002
Occupation 3	4.790	1.865	0.065	2.57	0.011

F = 134.41, *p* < 0.001, adjusted R2 = 0.735. MSE: maternal self-efficacy; EPDS: Edinburgh Postnatal Depression Scale; PSSS: Postpartum Social Support Scale.

## Data Availability

The data presented in this study are available on request from the corresponding author. The data are not publicly available due to privacy restrictions.
